# CT-ORG, a new dataset for multiple organ segmentation in computed tomography

**DOI:** 10.1038/s41597-020-00715-8

**Published:** 2020-11-11

**Authors:** Blaine Rister, Darvin Yi, Kaushik Shivakumar, Tomomi Nobashi, Daniel L. Rubin

**Affiliations:** 1 Department of Electrical Engineering, Stanford University, 350 Jane Stanford Way, Stanford, CA 94305 USA; 2 Department of Biomedical Data Science, Stanford University, 1265 Welch Road, Stanford, CA 94305 USA; 3 Department of Radiology, Stanford University, 300 Pasteur Drive, Stanford, CA 94305 USA

**Keywords:** Bladder, Liver, Skeleton, Kidney

## Abstract

Despite the relative ease of locating organs in the human body, automated organ segmentation has been hindered by the scarcity of labeled training data. Due to the tedium of labeling organ boundaries, most datasets are limited to either a small number of cases or a single organ. Furthermore, many are restricted to specific imaging conditions unrepresentative of clinical practice. To address this need, we developed a diverse dataset of 140 CT scans containing six organ classes: liver, lungs, bladder, kidney, bones and brain. For the lungs and bones, we expedited annotation using unsupervised morphological segmentation algorithms, which were accelerated by 3D Fourier transforms. Demonstrating the utility of the data, we trained a deep neural network which requires only 4.3 s to simultaneously segment all the organs in a case. We also show how to efficiently augment the data to improve model generalization, providing a GPU library for doing so. We hope this dataset and code, available through TCIA, will be useful for training and evaluating organ segmentation models.

## Abstract (summary)

**Table:** 

Measurement(s)	organ subunit • image segmentation • brain segmentation • anatomical phenotype annotation
Technology Type(s)	unsupervised machine learning • Manual • computed tomography • supervised machine learning
Factor Type(s)	human organ
Sample Characteristic - Organism	Homo sapiens

Machine-accessible metadata file describing the reported data: 10.6084/m9.figshare.13055663

## Background & Summary

Machine learning has the potential to transform healthcare by automating those tasks which defy mathematical description. In particular, fully-convolutional neural networks (FCNs) have far surpassed what was previously thought possible in semantic segmentation across all imaging domains^1,2^. These models rival human visual recognition on any task for which sufficient data are available. However, with limited training data these models will overfit, failing to generalize to unseen examples. This is particularly important for medical images, which are often expensive and laborious to annotate, even when the task is intuitive. One such task is detecting and segmenting organs in computed tomography (CT) images, which has wide applicability in treatment planning, morphology and computer-aided diagnosis. Despite the relative ease of locating various organs in the human body, precise annotation of organ boundaries is tedious and vague, presenting a serious obstacle to automated solutions.

Several CT organ segmentation datasets are already publicly available, including the SLIVER, Pancreas-CT, and Medical Decathlon collections^3–5^. Most of these datasets are limited to a single organ, and it is not possible to simply combine all the data together for multi-organ segmentation, since this requires consistent labeling of all objects of interest across all the images. Some existing datasets label multiple organs in every scan. The most popular of these are the one from Gibson *et al*. and the VISCERAL Anatomy3 dataset from Jimenez-del-Toro *et al*.^6,7^. While the Anatomy3 dataset offers an impressive range of organs and imaging modalities, it is limited to only 20 cases, far too few to train a model that will be robust in clinical practice where rare and anomalous conditions are frequently found. On the other hand, Gibson et. al provide 90 labeled cases, comprised of data from the Beyond the Cranial Vault and Pancreas-CT datasets^4,6,8^. However, these annotations are manually cropped around a specific region of the abdomen. This requires user interaction, and precludes the possibility of training a model which works on the whole body. A further limitation is that none of these datasets label the skeleton, which is an important site of metastatic cancer and other disease. All of these factors suggest the need for a new dataset encompassing multiple organs across the whole body with a large number of cases.

To address this need, we developed a new dataset consisting of 140 CT scans with six organ classes, which we call CT-ORG. We started from an existing dataset, the LiTS Challenge, which focuses on the liver, and significantly expanded it to encompass a wider range of organs and imaging protocols^2^. Our dataset contains volumetric labels for the liver, lungs, bladder, kidney, bones and brain. The data are divided into 119 training volumes and 21 testing volumes, which were annotated to a higher degree of accuracy for certain organs. The data exhibit a wide variety of imaging conditions collected from various medical centers, to ensure generalizability of the trained models. To our knowledge, this is the largest publicly available multi-organ dataset.

We developed several methods to annotate the data more efficiently. First, we segmented the lungs and bones using unsupervised morphological algorithms, which were accelerated using 3D Fourier transforms. This reduced annotation time to reasonable levels which would not be possible manually. We then manually refined and corrected the automatic labels to create a withheld test set of 21 volumes, which allows us to evaluate the accuracy of the morphological algorithms. The test set could serve as a useful benchmark for evaluating various organ segmentation methods, as it comprises a wide variety of organs, from the large and easily identifiable liver, to the small and discreet bladder.

To demonstrate the utility of our data, we trained a deep neural network to simultaneously segment all the labeled organs. We applied various data augmentations to improve model generalization. The model achieves high accuracy on the manually-annotated test set, processing a CT scan at 3 mm^3^ resolution in only 4.3 s. Dice scores on the test set range from 78–96% per organ, with an average of 90% across all organs. Our experiments show that the dataset suffices to train a deep neural network, which outperforms the morphological algorithms from which it was trained.

## Methods

This section describes the process of annotating the CT images with organ masks. Because computation was used to accelerate the process, we first describe the mathematical background, then proceed to the specific morphological algorithms used to segment the lungs and bones, and finally the manual annotation process used for all organs.

### Morphological segmentation

We used morphological algorithms to generate training data for the bones and lungs. This concept is called *weak supervision*, an active area of machine learning research. In the medical domain, weak supervision was previously exploited for brain ventricle segmentation^9^.

In what follows, we describe the basics of *n*-dimensional image morphology, and how we accelerated these operations using Fourier transforms. Then we describe the specific algorithms used to segment the lungs and bones.

### Morphology basics, acceleration by Fourier transforms

Let $f:{{\mathbb{Z}}}^{n}\to {{\mathbb{F}}}^{2}$ denote a binary image, and $k:{{\mathbb{Z}}}^{n}\to {{\mathbb{F}}}^{2}$ the structuring element. Then the familiar operation of morphological dilation can be written as

1 $$D(f,k)(x)=\left\{\begin{array}{cc}0, & (f\ast k)(x)=0\\ 1, & {\rm{otherwise}}.\end{array}\right.$$

That is, we first convolve *f* with *k*, treating the two as real-valued functions on ${{\mathbb{Z}}}^{n}$. Then, we convert back to a binary image by setting zero-valued pixels to black, and all others to white. Erosion is computed similarly: let $\bar{f}$ denote the binary complement of *f*; then erosion is just $E(f,k)(x)=\bar{D(\bar{f}\,,k)}$. Similarly, the opening and closing operations are compositions of erosion and dilation. Note that in actual implementation, it is more numerically stable to take $f\ast k < \epsilon$, for some small $\epsilon$, as a proxy for $f\ast k=0$. Also, for finite image volumes, the convolution must be suitably cropped so that $f\ast k$ has the same dimensions as *f*. This can be implemented by shifting the phase of the Fourier transforms and extrapolating the out-of-bounds values with 0 for $\widehat{f}$ and 1 for $\widehat{\bar{f}}$.

The advantage of writing dilation this way is that all of the basic operations in *n*-dimensional binary morphology reduce to a mixture of complements and convolutions. Complements are .inexpensive to compute, while convolutions can be accelerated by fast Fourier transforms, due to the identity $\widehat{f\ast k}=\widehat{f}\,\cdot \,\widehat{k}$, where $\widehat{f}$ denotes the Fourier transform of *f*. This allows us to quickly generate training labels by morphological operations, which is especially beneficial when the structuring elements are large. In what follows, we describe how morphology is used to generate labels for two organs of interest, the skeleton and lungs. These were chosen because their size and intensity contrast enable detection by simple thresholding.

### Morphological detection and segmentation of CT lungs

The lungs were detected and segmented based on the simple observation that they are the two largest air pockets in the body. The morphological algorithm is as follows:

1. Extract the air pockets from the CT scan by removing all voxels greater than *τ* = −150 Hounsfield units (HU). The resulting mask is called the thresheld image, denoted *f*_*τ*_.
2. Puncture the thin wall of the exam table by closing *f*_*τ*_ with a rectangular prism-shaped structuring element of width 1 × 10/*d* × 1 voxels, where *d* is the pixel spacing in mm. This connects the air inside the hollow exam table to the air outside the patient, assuming the usual patient orientation.
3. Remove any mask component that is connected to the boundary of any axial slice. This removes air outside of the body, as well as the hollow interior of the exam table, while preserving the lungs.
4. Remove the chest wall and other small air pockets by opening the image using a spherical structuring element with a diameter of 1 cm.
5. From the remaining mask, take the two largest connected components, which are almost certainly the lungs.
6. Finally, undo the effect of erosion by taking the components of *f*_*τ*_ which are connected to the two detected lungs.

Note that the final step is a form of morphological reconstruction, as elaborated in Vincent^10^.

**Fig. 1:**
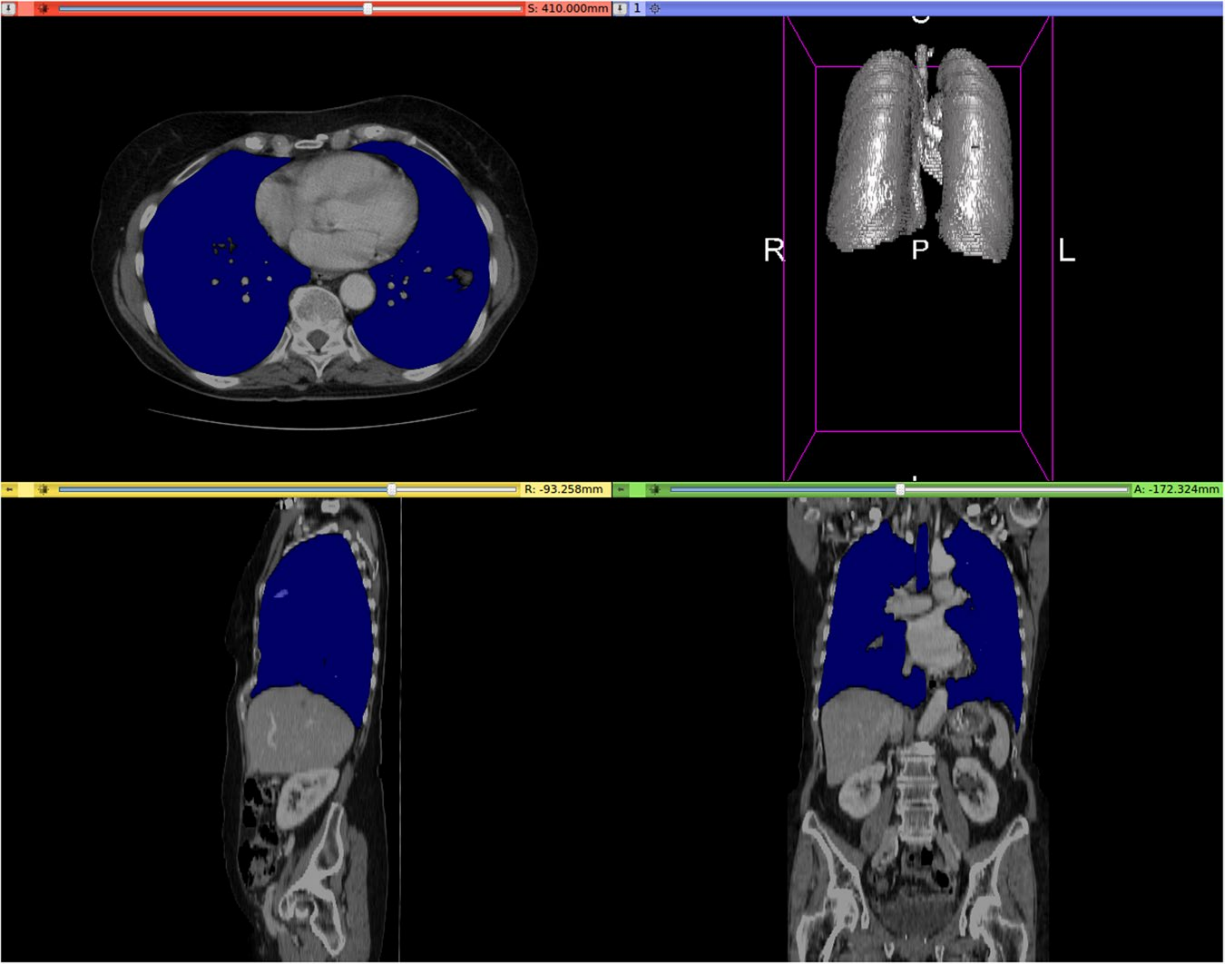
Example of CT lung detection and segmentation by image morphology. Lung mask overlaid in blue. Rendered by 3D Slicer^24^.

This algorithm is fairly robust, on both abdominal as well as full-body CT exams showing the full width of the exam table. The only major drawback is that the lungs are not separated from the trachea, which may not necessarily be an issue. See the Experimental results section for a quantitative evaluation. An example output is shown in Fig. 1. A quick web search reveals that similar algorithms have previously been used to segment the lungs, although not necessarily for the purpose of training a deep neural network.

### Morphological detection and segmentation of CT bones

Bone segmentation proceeds similarly to lung segmentation, by a combination of thresholding, morphology and selection of the largest connected components. This time we define two intensity thresholds, *τ*_1_ = 0 and *τ*_2_ = 200 HU. These were selected so that almost all bone tissue is greater than *τ*_1_, while the hard exterior of each bone is usually greater than *τ*_2_. The algorithm is as follows:

1. Threshold the image by *τ*_2_. This extracts the hard exteriors of the bones, but also inevitably includes some unwanted tissues, such as the aorta, kidneys and intestines, especially in contrast-enhanced CTs.
2. Select only the largest connected component from the thresheld image, which is the skeleton. This removes unwanted hyper-intense tissues which are typically not connected to the skeleton. It does have the drawback of excluding the ribs on abdominal images, in which they are disconnected from the rest of the skeleton. However, this drawback is acceptable for the purposes of generating training data.
3. Close the mask using a spherical structuring element with a diameter of 2.5 cm. This fills gaps in the cortical bone, which may be too thin to be seen in digital CT images.
4. Apply the threshold *τ*_1_ to remove most of this unwanted tissue between the bones, which might have been closed in the previous step.
5. For each *xy*-plane (axial slice) in the image, fill any holes not connected to the boundary. This fills in the centers of large bones, such as the pelvis and femurs, assuming the usual patient orientation.

**Fig. 2:**
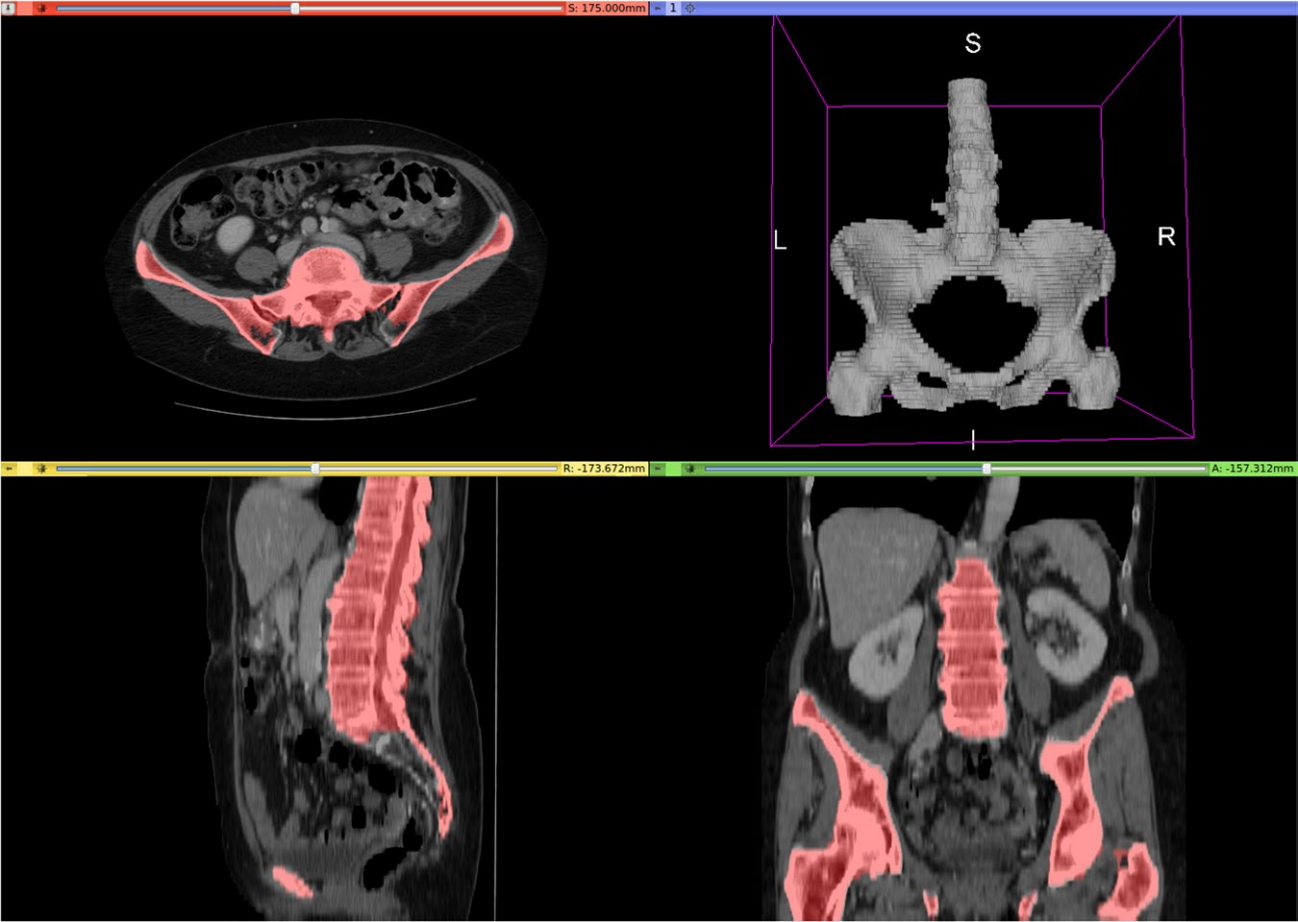
Example of CT skeleton detection and segmentation by image morphology. Skeleton mask overlaid in red. Rendered by 3D Slicer^24^.

This simple algorithm is sufficiently accurate to train a deep neural network, and serves as a useful basis for future manual refinement. The accuracy is evaluated quantitatively in the [subsec:experiment_CT-organ-segmentation]Experimental results section. See Fig. 2 for an example output, which omits some sections of the sacrum and pelvis that will need to be manually corrected in the testing set.

### Manual annotation

Morphological segmentation is sufficiently accurate to create training data, but manual correction is required for the more accurate testing set. Furthermore, segmentation based on thresholding is not suitable for soft tissues, which exhibit poor intensity contrast in CT. For these reasons, we annotated the remaining organs using the ITK-SNAP software^11^. The annotation process consisted of manual initialization, followed by active contour segmentation with user-adjusted parameters, and finally a manual correction using the 3D paintbrush tool. The liver, kidneys, bladder and brain were manually annotated in both the testing and training set. The lungs were manually annotated in the test set only. The bones followed the same basic procedure as the lungs, but we saved time on the initialization stage by starting from the output of the morphological algorithm, followed by manual refinement. Refining the bone masks consisted mostly of including small, disconnected bones such as the ribs and scapulae, and removing the spinal cord.

All manual annotations were made or overseen by a graduate student with several years’ experience annotating CT images. Following the initial annotation, the 21 cases in the test set were reviewed by an experienced board-certified radiologist, after which 9 out of 21 cases were refined to a higher degree of accuracy. The radiologist noted that the annotations were of high quality, as subsequent corrections were minor.

In the majority of cases, probably around 130 out of the 140 total, liver annotations were taken from the existing LiTS Challenge dataset^2^. We added our own liver annotations where the original dataset was missing them, as well as for new images not in LiTS. We included LiTS liver annotations in both the test and training set, as their methodology for manual annotation was very similar to ours.

### Human subjects

All imaging data was either publicly available, or collected from Stanford Healthcare. This study was approved by the relevant IRB.

## Data Records

All data are available on The Cancer Imaging Archive (TCIA) under the title *CT-ORG: CT volumes with multiple organ segmentations*^12,13^.

The dataset consists of 140 CT scans, each with five organs labeled in 3D: lung, bones, liver, kidneys and bladder. The brain is also labeled on the minority of scans which show it. Of the 140 total image volumes, 131 are dedicated CTs, the remaining 9 are the CT component taken from PET-CT exams. The dedicated CT images are derive from several clinical sites including Ludwig Maxmilian University of Munich, Radboud University Medical Center of Nijmegen, Poly-technique & CHUM Research Center Montreal, Tel Aviv University, Sheba Medical Center, IRCAD Institute Strasbourg and Hebrew University of Jerusalem^2^. The PET-CT images all derive from Stanford Healthcare.

Each image was taken from a different patient. The gender distribution is 62.5% male and 37.5% female. While the ages of most patients could not be determined, they should be considered representative of typical liver cancer patients. Patients were included based on the presence of lesions in one or more of the labeled organs. Most of the images exhibit liver lesions, both benign and malignant. The number of distinct liver lesions per patient varies from 0 to 75, and the volume of lesions varies from 38 mm^3^ to 349 mm^3^. Some also exhibit metastatic disease derived from cancer in the breast, colon, bones and lungs. The axial resolution of dedicated CTs varies from 0.56 mm to 1.0 mm. Images derive from pre- and post-treatment stages, with and without contrast enhancement. See Bilic *et al*. for more information^2^.

All files are stored in NIfTI-1 format with 32-bit floating point data.

Images are stored as “volume-*x*.nii.gz” where *x* is the one- to three-digit case number, ranging from 0–139. All images are CT scans, under a wide variety of imaging conditions including high-dose and low-dose, with and without contrast, abdominal, neck-to-pelvis and whole-body. Many patients exhibit cancer lesions, especially in the liver, but they were not selected according to any specific disease criteria. Numeric values are in Hounsfield units.

The first 21 volumes (case numbers 0–20) constitute the testing split. The remaining volumes constitute the training split. Training masks suffice for training a deep neural network, but should not be considered reliable for evaluation.

Segmentations are stored as “labels-*x*.nii.gz”, where *x* is the same number as the corresponding volume file. Organs are encoded as 32-bit floating point numbers according to Table 1.

**Table 1:** Organ labeling scheme.

Organ class	Label
Background	0
Liver	1
Bladder	2
Lungs	3
Kidneys	4
Bone	5
Brain	6

## Technical Validation

To confirm the utility of the dataset, we trained an FCN to segment the labeled organs, and evaluated the model performance on our test set. This model was chosen to represent a broad class of deep learning methods which are the current state of the art in organ segmentation^2,14–17^. Our experiments confirm that the training data are sufficiently numerous for the model to generalize to unseen examples, and that the morphological segmentations of bones and lungs are sufficiently realistic to yield acceptable performance when compared to manual organ annotations. We also directly evaluate the similarity between morphological segmentation and the ground-truth manual annotations.

### Neural network architecture

This section describes the design and training of our predictive model; both the inputs and outputs, pre- and post-processing, the neural network itself, and the training loss function.

### Pre- and post-processing

Our neural network takes as input a 120 × 120 × 160 image volume, and outputs a 120 × 120 × 160 × 6 probability map, where each voxel is assigned a class probability distribution. As usual, we take the the argmax probability for each voxel to convert the six probabilities to a single class label. Some works have addressed the issue of limited viewing resolution by training multiple FCNs, each working at different scales^15^. However, we found that a single resolution achieves reasonable performance. To reduce memory requirements, we resample all image volumes to 3 mm^3^. Resampling uses Gaussian smoothing as a lowpass filter to avoid aliasing artifacts, followed by interpolation at the new resolution. Since each CT scan has its own millimeter resolution for each dimension *u* = (*u*_1_, *u*_2_, *u*_3_), we adjust the Gaussian smoothing kernel according to the formula $g(x)\propto {\rm{\exp }}(-{\sum }_{k=1}^{3}{x}_{k}^{2}/{\sigma }_{k}^{2})$ where the smoothing factors are computed from the desired resolution *r* = 3 according to ${\sigma }_{k}=\frac{1}{3}{\rm{\max }}(r/{u}_{k}-1,0).$ This heuristic formula is based on the fact from digital signal processing that, in order to avoid aliasing, the cutoff frequency should be placed at *r*/*u*_*k*_, the ratio of sampling rates, on a [0,1] frequency scale.

After processing the resampled input with the neural network, we resample the 120 × 120 × 160 prediction map back to the original image resolution by nearest neighbor interpolation. One difficulty with this scheme is that CT scans vary in resolution and number of slices, and at 3 mm^3^ we are unlikely to fit the whole scan in our network. For training, we address this by selecting a 120 × 120 × 160 sub-region from the scan uniformly at random. For inference, we cover the scan by partially-overlapping sub-regions, averaging predictions where overlap occurs. We distribute the tiles across all available GPUs to process them in parallel.

### Model

We chose a relatively simple model which balances speed and memory consumption with accuracy. Our model is essentially a three dimensional U-Net, which is to say an FCN divided into *chunks* which are separated by either pooling or upsampling, where chunk *k* has a skip connection to chunk $N-k+1$, i.e. the first chunk is connected to the last, the second to the second-from-last, etc. This is probably the most popular model category for semantic segmentation, which we defer to the literature for explanation^1,14,16,17^. For a more advanced variant, see the DeepLab family of models which utilize separable convolutions at various scales^18^.

Each of our U-Net chunks contains two convolution layers and either a max-pooling or “transposed convolution” (also called deconvolution) upsampling layer. Decimation chunks are *followed* by pooling, while interpolation chunks are *preceded* by upsampling. All of our convolution kernels have 3 × 3 × 3 dimensions, while our max-pooling layers use 2 × 2 × 2 windows. All convolutions are followed by constant “bias” addition, batch normalization and rectification^19^. All layers have a stride of one voxel. The only significant departure for volumetric imaging is the considerable memory requirement. To save memory, we use fewer feature maps per layer. The parameters of each chunk are enumerated in Table 2. The final chunk is followed by a convolution layer outputting six feature maps, one per object class, which are then passed into a softmax layer yielding the output probabilities.

**Table 2:** List of chunks in our fully-convolutional neural network.

Chunk Type	Output channels
Decimation	32
	64
	64
	128
Interpolation	64
	64
	32

Each chunk consists of three layers.

Counting either convolution or pooling as one layer, our model contains 22 layers in total, three per chunk and one for the final convolution. The number and width of the layers was chosen heuristically to fit in the 12 GB of memory available to each of our graphics cards. No special techniques were used to reduce memory consumption, such as checkpointing or reducing the arithmetic precision^20^. We explored checkpointing, but abandoned it as the memory savings were equal to the increase in computation time.

### Training loss

Most tasks in medical image segmentation exhibit extreme class imbalance, where the vast majority of voxels are members of the “background” class. Furthermore, in multi-organ segmentation we must differentiate between objects of vastly different sizes, ranging from the bladder on one extreme to the lungs on the other. To address this class imbalance, we employed the IOU loss, a variant of the popular Dice loss proposed by Milletari *et al*.^14^. The IOU loss attempts to minimize the intersection over union (IOU) of the output segmentation, also called the Jaccard index. Since the IOU depends on binary values, it cannot be optimized by gradient descent, and thus it is not directly suitable as a loss function for deep learning. Instead, our strategy is to define a smooth function which is equal to the IOU when the output probabilities are all either 0 or 1. Since ${\{0,1\}}^{n}\subset {[0,1]}^{n}$, we consider the training labels *y* as a vector in [0,1]^*n*^ consisting only of probabilities 0 and 1. Then, we define the smooth IOU loss as

2 $${\mathcal{L}}=1-\frac{p\cdot y}{{\left\Vert p\right\Vert }_{1}+{\left\Vert y\right\Vert }_{1}-p\cdot y}$$

where ${\left\Vert p\right\Vert }_{1}={\sum }_{k}pk$, since ${p}_{k}\ge 0$ for all *k*.

The binary version of the IOU loss function was previously proposed by Rahman and Wang^21^. To extend this to multi-class segmentation, we simply average ${\mathcal{L}}$ for each class, including the background. This approach seems simpler and more naturally motivated than the multi-class Dice scheme of Sudre *et al*.^22^.

## Experimental Results

In this section we describe our experiments validating the suitability of our data for deep learning tasks. We trained the previously-described neural network both with and without data augmentation, evaluating its performance against unseen manual annotations. We also evaluated the accuracy of the morphological segmentation algorithms.

As is typically the case in deep learning, we used data augmentation to expand our dataset beyond what was manually annotated. We randomly generated additional training data from the 119 original volumes, by applying known transformations to both the image and the training labels. This typically reduces the generalization error of the model, and promotes robustness to expected geometric and photometric transformations, especially when applied to smaller datasets. We applied a variety of transformations including affine warping, intensity windowing, additive noise and occlusion. These operations were accelerated by the GPU with our publicly-available CUDA library, as described in the Code availability section.

We implemented our model in TensorFlow according to the design decisions in the Model section^23^. We used fairly standard choices for hyperparameters and training procedure. Our loss function was optimized by the RMSProp algorithm, along with an added ${\ell }_{2}$ regularization term of weight *λ* = 10^−7^. The learning rate began at *α* = 10^−3^, with no data augmentation. During each training iteration we selected 3 separate cases uniformly with replacement, extracting a 120 × 120 × 160 volume from each to form the training batch. We used three GPUs for training and one for data augmentation, with a single volume assigned to each GPU. After 15666 training iterations we decreased *α* to 10^−4^ and enabled data augmentation. Then we trained on randomly-generated volumes for an additional 32582 iterations. These parameters were selected heuristically to maximize performance, as training took more than one day on our server, prohibiting exhaustive search.

**Fig. 3:**
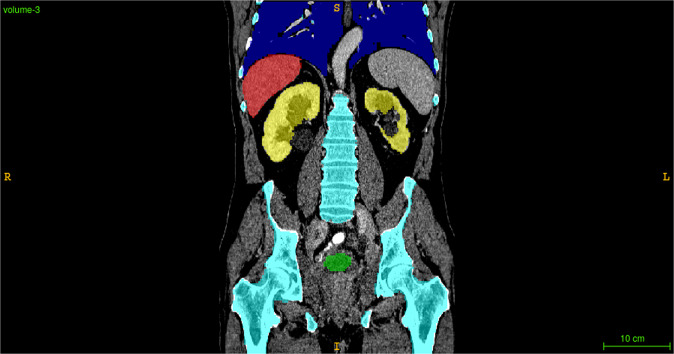
Example neural network prediction on the unseen test set.

To robustly estimate the performance on unseen data, we averaged the Dice scores across all classes for each case, then selected the median score across all checkpoints from the pre- and post-data augmentation phases, possibly removing the first score to ensure the median is achieved. Finally, we took the per-class scores corresponding to the earliest median example for each phase. We then repeated this procedure for the mean symmetric surface distance and Hausdorff distance, possibly choosing a different representative iteration for each metric. We excluded the brain class from evaluation, as it is present in only 8 out of 119 training volumes, so the model had trouble learning to distinguish it when trained with uniform sampling. Inference took an average of 4.3 s per CT scan using all four GPUs. The results are shown in Tables 3–5. See Fig. 3 for an example prediciton on an unseen test case.

**Table 3:** Dice scores, mean (± standard deviation) per case over the test set.

MethodData augmentation	Neural network		Morphologyn/a
	No	Yes	
Lung	93.8 ± 5.9	**95.5** ± 4.5	93.6 ± 12.5
Liver	92.0 ± 3.6	**95.2** ± 2.5	n/a
Bone	82.7 ± 7.6	85.8 ± 6.2	**86.0** ± 6.1
Kidney	88.2 ± 7.9	**91.8** ± 4.0	n/a
Bladder	58.1 ± 22.3	**77.7** ± 17.8	n/a

Median performance from training checkpoints taken every 50 iterations.

**Table 4:** Mean symmetric surface distance.

MethodData augmentation	Neural network		Morphologyn/a
	No	Yes	
Lung	1.93 ± 3.16	**1.63** ± 2.85	4.66 ± 2.58
Liver	1.21 ± 1.55	**1.09** ± 1.19	n/a
Bone	0.95 ± 0.42	**0.92** ± 0.44	4.55 ± 8.53
Kidney	1.36 ± 0.98	**0.51** ± 0.21	n/a
Bladder	**5.20** ± 12.4	6.25 ± 14.0	n/a

Compare to Table 3.

**Table 5:** Hausdorff distance. Compare to Table 3.

MethodData augmentation	Neural network		Morphologyn/a
	No	Yes	
Lung	59.8 ± 75.2	**35.2** ± 31.5	57.9 ± 42.9
Liver	40.5 ± 23.1	**32.8** ± 43.0	n/a
Bone	29.9 ± 9.55	**29.8** ± 12.1	153.9 ± 67.8
Kidney	28.0 ± 27.2	**17.3** ± 20.5	n/a
Bladder	**16.5** ± 28.3	29.9 ± 47.5	n/a

### Discussion of experimental results

These results show that our data are sufficient to train a multi-organ segmentation model to a level of accuracy which could be clinically useful. They also show that our methodology of generating training data with unsupervised algorithms is sufficient to train a deep neural network for certain organs. Finally, they show that the unsupervised algorithms reasonably agree with manual annotations from the test set. Indeed, the last of column of Table 3 suggests that the morphological bone and lung segmentations agreed closely with the manual corrections.

While 140 cases is fewer than typical for deep learning, our model still avoids overfitting because organs are segmented on a per-voxel basis. Compared to classification models which output a single label per image, segmentation models typically succeed with fewer data. This is especially true when using data augmentation, which changes the labels as well as the image content.

In our experiments, data augmentation improved the Dice score for all organs. It also improved the surface distances, with the exception of the bladder. This suggests that the training dataset of 119 cases is not yet large enough for peak performance, and that some overfitting occurs when the data are not augmented. Future improvements could be had by expanding our dataset, or combining it with existing ones. The main limitation in combining datasets is that we can only reliably predict those organs labeled in all data sources.

Interestingly, the neural network outperformed the morphological lung segmenter by all metrics, and outperformed the bone segmenter in all except for the Dice score, in which it was nearly equal. This closely matches the results of Ghafoorian *et al*., in which a neural network surpasses the unsupervised algorithm from which it is trained^9^. For organ segmentation, the main disadvantage of morphology is that it is prone to catastrophic failure in surprising ways. For example, the morphological lung segmenter considers both lungs as belonging to the same object if they are sufficiently close together, instead detecting some abdominal air pocket as the remaining lung. Similarly, the bone segmenter fails to detect the ribs if the scan is cut off before they meet the spine, and sometimes fails to connect bones across large joints such as the shoulder. These issues are likely correctable with more complex hand-engineered rules, or on a case-by-case basis with manual adjustment of parameters. In contrast, a neural network captures the visual appearance of each organ, which is often more reliable than the shape or number of objects. When segmenting a large variety of organs, a single network has obvious conceptual and computational advantages over a litany of brittle, organ-specific solutions.

## Usage Notes

The data are suitable for visualization in a variety of software, including 3D Slicer and ITK-SNAP^11,24^. For best results, users should take into account the resolution of each volume, which is stored in the NIfTI file headers, resampling the data accordingly. At higher resolutions, the images will likely need to be cropped and processed in tiles, as described in the Pre- and post-processing section. We found that model performance was consistently improved by training data augmentation, as elaborated in the Data transformations section. See the Code availability section for our data augmentation code and pre-trained model.

## Data Availability

All code is available on Github for our morphological segmentation, GPU data augmentation, and pre-trained model. The pre-trained model is packaged in a Docker container for ease of use. (Please see https://github.com/bbrister/ctOrganSegmentation, https://github.com/bbrister/cudaImageWarp, https://github.com/bbrister/ctorgansegdocker and https://hub.docker.com/repository/docker/bbrister/organseg.)
